# *In-situ* Raman analysis of hydrogenation in well-defined ultrathin molybdenum diselenide deposits synthesized through vapor phase deposition

**DOI:** 10.1038/s41598-020-67132-0

**Published:** 2020-06-23

**Authors:** Peter Joseph Santiago, Francisco Ramirez, Hadi Tavassol

**Affiliations:** 1 Department of Physics and Astronomy, California State University, Long Beach, United States; 2 Department of Chemistry and Biochemistry, California State University, Long Beach, United States

**Keywords:** Materials chemistry, Surface chemistry, Materials for energy and catalysis

## Abstract

We report on the synthesis, characterization and *in-situ* Raman spectroscopy analysis of hydrogenation in ultrathin crystalline MoSe_2_ deposits. We use a controllable vapor phase synthesis method using MoSe_2_ powder as the only precursor, to fabricate nano- to micro-size few layer thick MoSe_2_ deposits with tunable number densities on SiO_2_/Si substrates. We employ this controllable synthesis method to correlate characteristic Raman spectroscopy response of MoSe_2_ at *ca*. 242 cm^−1^ (A_1g_) and *ca*. 280 cm^−1^ (E_2g_^1^) with the thickness of the deposits acquired from atomic force microscopy (AFM). We also use this array of well-defined atomically thin MoSe_2_ deposits to study possible hydrogenation effects on select architectures using *in-situ* Raman spectroscopy. Interestingly, our analysis indicates that ultrathin MoSe_2_ deposits with exposed edges show a blue shift of 1–2 cm^−1^ when exposed to H_2_ flow at 150–250 sccm for 2–4 hours in a sealed reaction cell. Exposure to Ar flow under same condition reverses the observed shift in the A_1g_ mode of the select MoSe_2_ deposits. Our measurements provide *in-situ* evidence for hydrogen adsorption on MoSe_2_ deposits at room temperature and insight into the possible active sites for hydrogen reactions on layered dichalcogenides at lower dimensions.

## Introduction

Low dimensional layered materials show many interesting and unexpected physical and/or chemical properties^1,2^. Dichalcogenide type materials are particularly interesting, since due their asymmetry they show increased interactions at low dimensions^3,4^. MoSe_2_, a layered dichalcogenide, is of interest due to its unique electrical, optical and catalytic properties^5–7^. MoSe_2_ has a direct band gap of *ca*. 1.5 eV, which is suitable for single-junction solar cells or photoelectrochemical devices^8,9^.

Chemical vapor deposition (CVD) growth of layered materials, including MoSe_2_, has been used to fabricate high quality ultrathin films^6,10^ which is essential in device fabrications. CVD growth of MoSe_2_ proves to be more challenging than the more widely studied MoS_2_ due to the lower chemical reactivity of Se compared to sulfur^11^. Here we use a custom vapor phase CVD method to fabricate scalable ultrathin MoSe_2_ films on SiO_2_/Si substrates. We use micro-Raman spectroscopy and atomic force microscopy for detailed analysis of thickness and size dependent spectroscopic response of CVD grown MoSe_2_. Vibrational spectroscopy, e.g. Raman spectroscopy is an effective characterization method for layered materials and correlates with size and thickness of deposits^12^, edge and grain boundary effects^13,14^, and chemisorption and physisorption of active species^15–17^.

Reactivity of the dichalcogenide materials increases at edge sites and lower dimensions^15,18^. Elucidation of the nature of the reactive sites in these materials is an important fundamental question and greatly influences their application. Reactivity of the dichalcogenide type materials is thought to be limited to the edge sites^15,18^ and/or metallic phases^19^. Indeed defect sites at the basal plane also show reactivity^20,21^, although high concentration of defects changes the chemistry of the material.

One of the early examples of edge reactivity was observed for hydrogen treated MoS_2_ nano clusters exposed to thiophene at elevated temperatures using scanning tunneling microscopy (STM)^15^. Later, theoretical work shed light into the optimum energetics of hydrogen adsorption at MoS_2_ edges which in fact mimic highly efficient Pt and enzymatic systems^22^. Edge site reactivity in MoS_2_ has been shown by electrochemical deposition and post mortem analysis, which are only evident at edge sites of the triangle shaped deposits^20,23^.

Understanding the initial stages of hydrogen adsorption on dichalcogenide type layered materials at lower dimensions is important in explaining reaction mechanism and pathways of hydrogen reactions. Hydrogen evolution reaction, hydrotreating, and formation of simple hydrocarbons from CO_2_ are initiated and subsequently advance through hydrogen activation on catalysts as the primary step. These reactions are particularly important in the quest for the production of renewable chemical fuels which is an attractive strategy in achieving carbon neutrality in coming decades^24^. Edge sites of Mo dichalcogenides are also intriguing due to their similarities to the active sites of nitrogenases which are highly efficient toward hydrogen reactions^22^.

The reactivity of low dimensional layered materials such as graphene and dichalcogenide type materials, *e.g*. MoSe_2_ studied here will determine their applicability in different devices. Low reactivity (low defect density) for graphene used in electrical devices is critical. The selective activity of asymmetric layered materials such as dichalcogenide for use in optical and/or electrochemical devices is also of great interest in their application.

Vapor-phase synthesis of dichalcogenides are particularly attractive, since they provide a direct and simple synthesis method for making high purity materials^25,26^. Here we present a simple and scalable vapor phase method to synthesize few layer thick MoSe_2_ on SiO_2_/Si (100) substrates. We use this method to make an array of deposits with different sizes, thicknesses and number densities of MoSe_2_ deposits. Particularly, we use *in-situ* Raman spectroscopy analysis of these well-defined MoSe_2_ deposits and show that a subset of ultrathin MoSe_2_ deposits with exposed edges show a blue shift in the A_1g_ feature of MoSe_2_ deposits upon exposure to H_2_ (g). Results presented here provide insight into the hydrogenation of dichalcogenide type materials, which are important in fundamental understanding of reactivity and application of these materials.

## Result and Discussion

### Vapor-phase synthesis of few-layer thick MoSe_2_ films

Well-defined MoSe_2_ films with varying sizes and number densities were deposited on SiO_2_/Si substrates using vapor-phase chemical vapor deposition (CVD). A custom CVD chamber in which temperature and gas environment is controlled was developed and used. Synthesis was performed at around 850 °C using high purity MoSe_2_ powder as the only precursor, under a controlled H_2_(g) environment with flow rates varying in the 20–100 sccm range for 10–20 min. More details and diagrams of the synthesis method are included in the supporting information. Synthesis of MoSe_2_ is more challenging than the widely studied MoS_2_, due to difference in chemical reactivity^27^. Vapor phase synthesis of MoSe_2_ from MoSe_2_ powder is particularly challenging, due to difficult formation of large, ultrathin MoSe_2_ deposits and easy formation of oxides upon incomplete nucleation and growth. To the best of our knowledge, the current work introduces a novel vapor-phase synthesis method for well-defined triangles of atomically thin MoSe_2_, where no Se and/or MoO_3_ precursors are used. Here, by controlling the synthesis chamber atmosphere prior and during the synthesis, we were able to synthesize and scale the size and density of ultrathin MoSe_2_ deposits.

**Figure 1:**
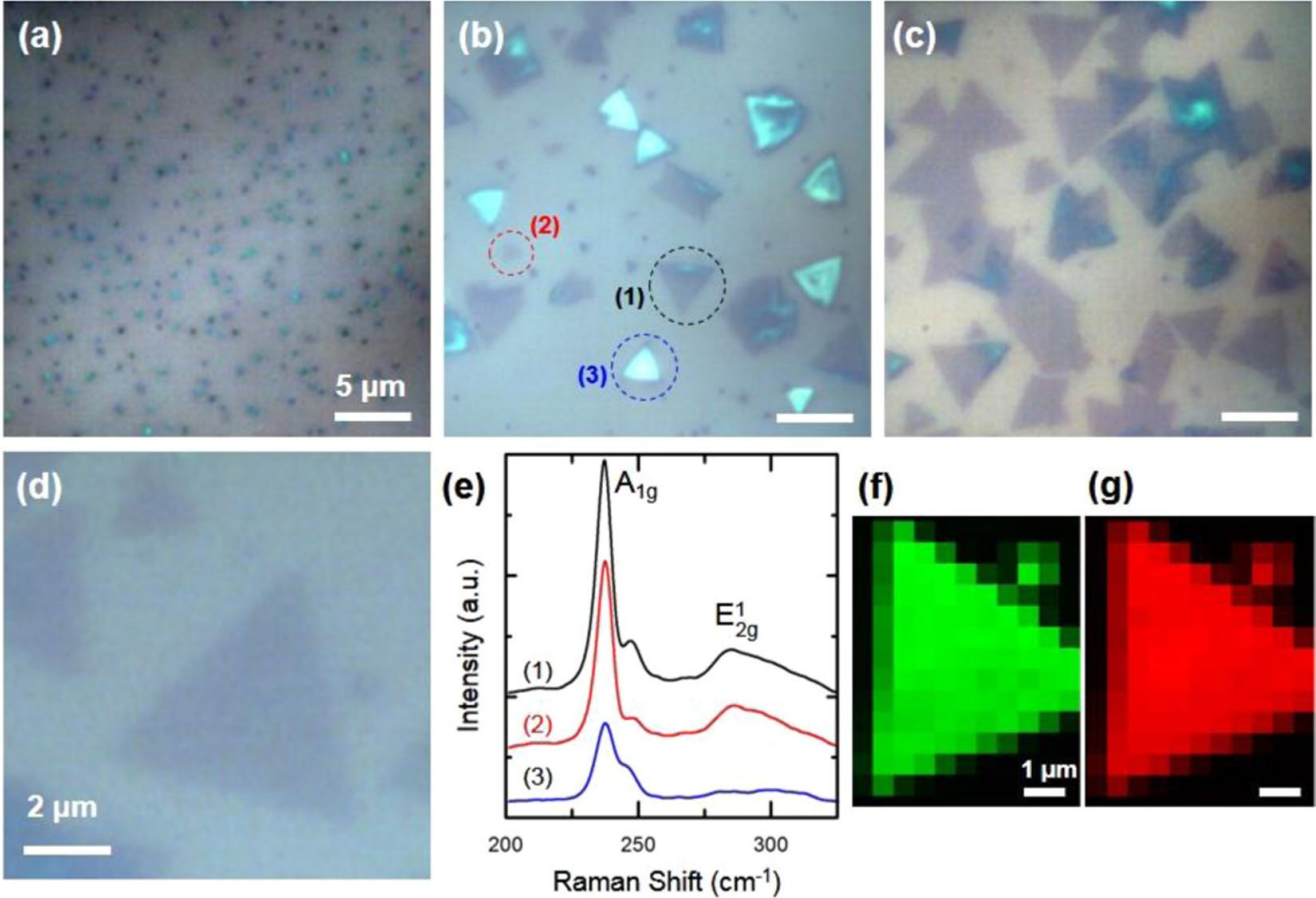
Optical images of the MoSe_2_ triangle synthesized using a vapor phase growth with varying H_2_ gas flow rates of (**a**) 20 sccm, (**b**) 75 sccm, and (**c**) 100 sccm. (**e**) Raman spectroscopy analysis of the deposits, shown in part (**b**), showing different brightness and sizes. (**f**) A_1g_ and (**g**) E^1^_2g_ mapping of the deposit shown in part (**d**), black in the mapping images show ca. 520 cm^−1^ peak of Si. The deposit shown in part (**d)** was deposited at 100 sccm H_2_ gas flow.

Density and size of atomically thin deposits were controlled by adjusting the deposition parameters, *i.e*. temperature and gas environment during synthesis. Figure 1 shows optical images, Raman analysis and Raman mapping of MoSe_2_ nanocrystals where size and number density of the deposits are controlled by adjusting synthesis conditions. Figure 1(a–c) show optical images of SiO_2_/Si substrates following synthesis at 800 °C for 20 min under 20, 75, 100 sccm of H_2_ gas respectively. Prior to the deposition, the chamber containing the substrates are ramped up to the desired temperature under Ar atmosphere. Size and number density of deposits is controlled by H_2_ (g) flow rate, where increasing the flow rare at the deposition temperature of 850 °C results in larger triangle shaped deposits. This observation agrees with reports showing similar trends between particle sizes and flow rate of the carrier gas in an ambient pressure CVD growth^27^. As shown in Fig. 1, we have developed a scalable and controllable method for making highly crystalline and ultrathin MoSe_2_ deposits.

Figure 1e shows Raman analysis of MoSe_2_ deposits highlighted in the optical image shown in Fig. 1b. Raman spectroscopy of the highlighted deposits agree with previous reports where two main features of A_1g_ at *ca*. 240 cm^−1^ and E_2g_^1^ at *ca*. 280 cm^−1^ are present^12,28^. The Davydov splitting explains the energy shift from 240.2 cm^−1^ to 242 cm^−1^ as number of layers increases^12,29^.

Raman analyses (Fig. 1e) also show that relative intensity of these two features correlate with their observed contrast in their optical images (Fig. 1b). Deposits (1) and (2) in Fig. 1b which appear darker in the optical images show more intense Raman features. Deposit (3), which appears bright in the optical image show lower intensity for the A_1g_ feature, and E_2g_^1^ feature is not present in the Raman spectrum. Figure 1d shows a higher magnification of a dark triangular shaped deposit of MoSe_2_ with well-defined edge sites. Figure 1f,g show Raman mapping of A_1g_ and E_2g_^1^ features corresponding to the deposit shown in Fig. 1d. Raman mappings of the two main features A_1g_ and E_2g_^1^ show a uniform intensity across the deposit. Larger area mapping of the A_1g_ peak of the triangle shaped features is shown in Fig. S2, confirming uniformity of the Raman signature across the deposits as well as the correlation with the contrast observed in the optical images. Previous reports have also shown the correlation of optical images with the Raman intensity, where thickness of exfoliated films correlate with intensity and higher number of MoSe_2_ layers show lower intensity in the Raman spectra^12,28^. Later, we will discuss our atomic force microscopy (AFM) analysis to further clarify the effect of the thickness of vapor phase CVD grown deposits on their optical and Raman response.

### Interdependence of thickness and Raman spectroscopy response

Changes in the chemical and electrical properties of atomically thin dichalcogenides including MoSe_2_ as a function of thickness is of great interest to their application in optical, electrical, and electrochemical systems. Detailed AFM and Raman analysis are used to further analyze the MoSe_2_ deposits synthesized here.

**Figure 2:**
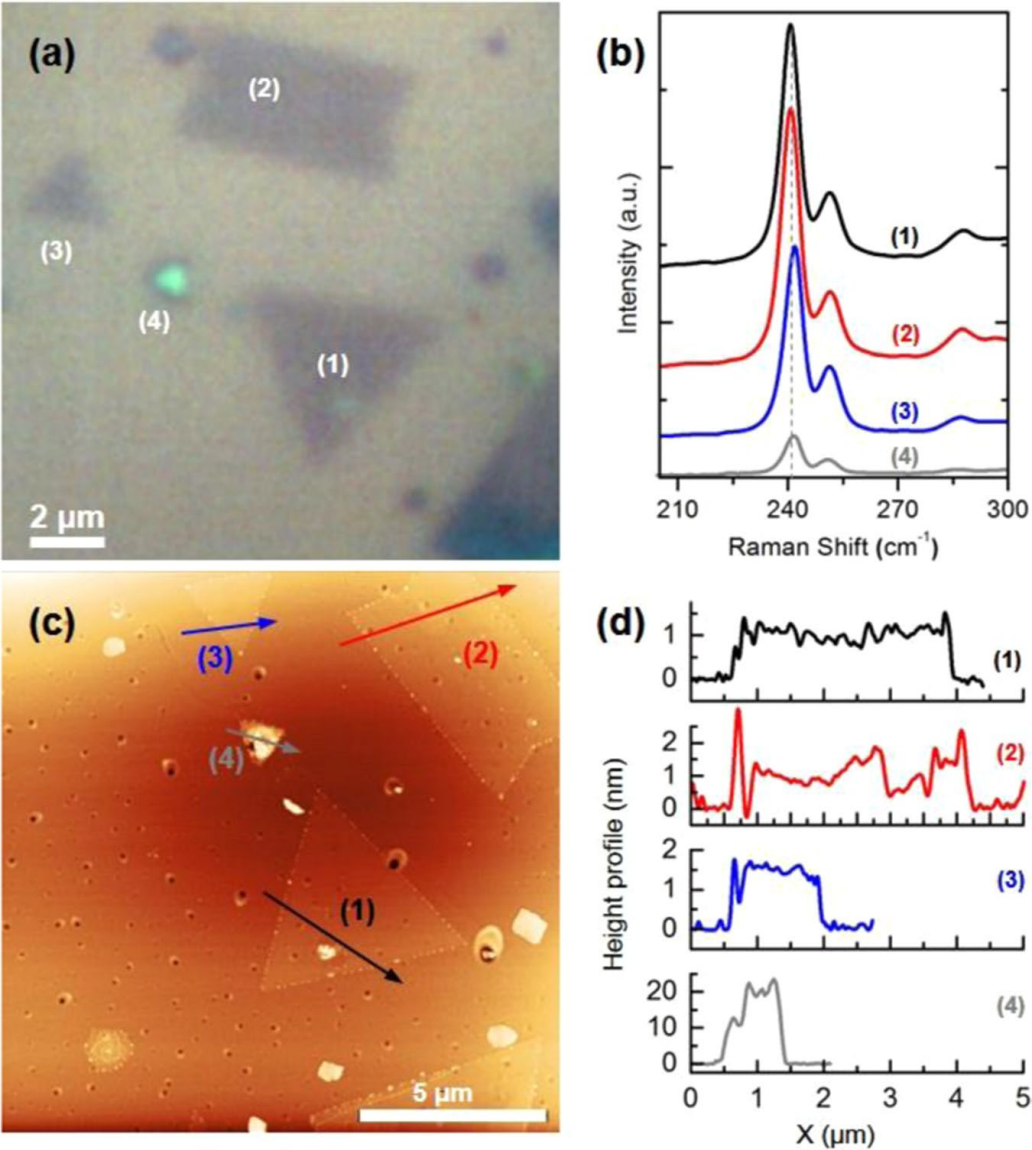
(**a**) Optical image of a MoSe_2_ deposits prepared using the vapor phase synthesis, (**b**) Raman spectroscopy analysis of different regions exhibited, (**c**) AFM image of the same region observed in the optical image and analyzed in the Raman, and (**d**) height profiles of the features shown in the AFM and optical image.

Figure 2a shows the optical image of the MoSe_2_ deposits synthesized using the method detailed above. The optical image labels four (1–4) triangular- and rectangular- shaped deposits with sizes of *ca*. 1–6 μm. Deposit (4) appears brighter than other deposits in the optical image. Figure 2b shows the Raman spectroscopy analysis of the deposits shown in part (a). The main two main features of A_1g_ at *ca*. 240 nm and E_2g_^1^ at *ca*. 280 nm are present as discussed before. Interestingly, there are changes in the intensity and energy of the main features. Deposits (1) and (2) show the highest intensity for both A_1g_ and E_2g_^1^ features. (3) and (4) show progressively lower intensities. The E_2g_^1^ feature is not present in the Raman spectrum of deposit (4). Additionally, a blue shift of *ca*. 1.8 cm^−1^ is present from deposit (1) to (4). Figure 2c shows the AFM image of the same deposits. A 10 μm × 10 μm area was scanned for this analysis. All the MoSe_2_ deposits show sharp edges and well-defined features which agree with the optical image shown in Fig. 2a. The growth of the dichalcogenides materials, including MoSe_2_, is thought to be limited by the nucleation which determines the quality of dichalcogenide type films^1,30^. Nucleation is thought to be only limited to defect sites^6,31^_._ Our optical images show micron size deposits, AFM analysis shows regularly shaped MoSe_2_ deposits formed following the nucleation on the SiO_2_ substrate.

Figure 2d shows the height profile of the deposits at the indicated line scans. MoSe_2_ monolayer thickness is *ca*. 0.7–1.0 nm^6,10,13^. AFM analysis (Fig. 2c,d) show thicknesses of as low as 1 nm in the deposited samples, pointing to the formation of mono, double and few layers thick MoSe_2_ deposits. The height profile shows well-defined edges, and uniform thickness across the deposit. Interestingly, the brightness of the deposits, intensity of the Raman features, and their corresponding Raman shift, correlate with the height of the deposits from AFM analysis. As the thickness of the deposits increases the Raman intensity decreases and the blue shift of *ca*. 1.8 cm^−1^ appears in the A_1g_ feature.

In addition to the thickness effect, another interesting aspect of few layer thick dichalcogenide films is the changes in the optical and electrical properties as well as reactivity of these materials between basal and edge sites where symmetry is broken. Here we use well defined MoSe_2_ deposits to study their reactivity changes.

## ***In-situ*** analysis of hydrogen interactions at lower dimensions

Reactivity of the layered materials under different conditions and toward select reactions is of great interest to their application in advanced devices. Dichalcogenides are particularly attractive, since they have shown to be very active toward hydrogen reactions^18,32,33^. The active sites of the layered materials from dichalcogenide types are relevant to many applications including electrical, optical, and electrochemical devices. Layered materials such as graphene, have been shown to be inactive at their basal planes, and only the structural imperfections show activity^21^.

Active sites of MoSe_2_ for hydrogen reaction catalysis are of interest^34,35^, although not as well studied as MoS_2_. *In-situ* analysis of well-defined ultrathin MoSe_2_ deposits synthesized here under different gas environments will provide insight into their reactivity and primary steps of catalyst activation. Hydrogen reactions are particularly important since hydrogen oxidation and hydrogen evolution reactions (HER) are key in water splitting and hydrogenation of CO, which are critical for production and use of chemical fuels. Hence, we employed a reaction cell to perform an *in-situ* Raman spectroscopy analysis of well-defined MoSe_2_ deposits under reactive, H_2_(g) and inert, Ar (g) conditions.

**Figure 3:**
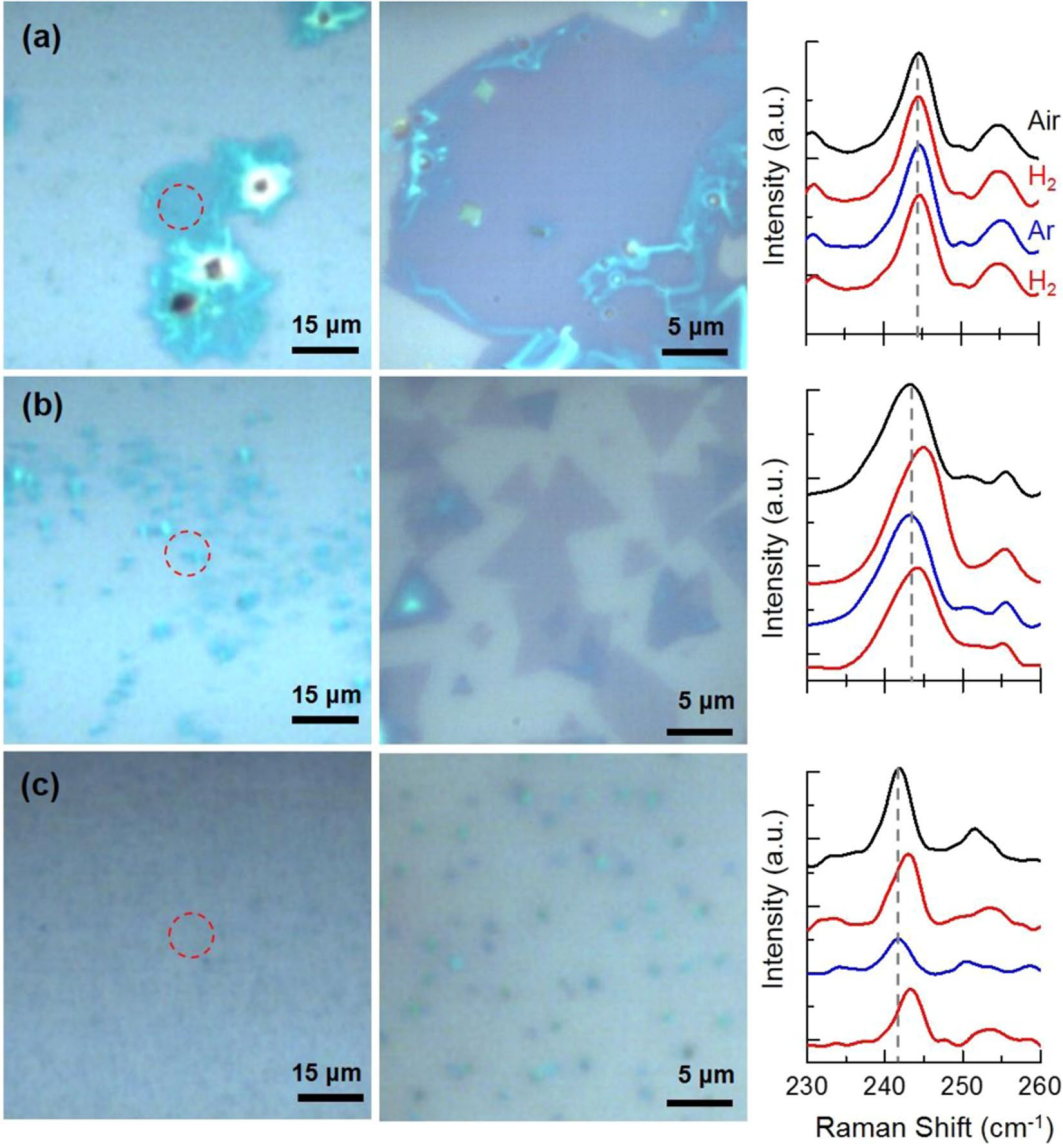
*In-situ* analysis of different MoSe_2_ deposits with varying sizes as the overhead atmosphere of the corresponding sample are saturated from air to H_2_, Ar and H_2_. (**a**) a large deposit of MoSe_2_ with no edge site in the probed area, (**b**,**c**) are deposits with varying sizes and number densities on the substrate.

For these studies, we use *in-situ* micro-Raman spectroscopy on films with MoSe_2_ deposits with different morphologies, sizes and number density on the substrate surface to gain insight into the reactivity of different architectures. Micro-Raman analysis was performed on different MoSe_2_ deposits under consecutive streams of Ar (g) and H_2_(g) in a sealed custom-made optical cell to monitor possible changes in the spectroscopic response of MoSe_2_ during reaction conditions. Figure 3 shows the optical images and the corresponding micro-Raman analysis of MoSe_2_ deposits. Micro-Raman analysis was performed on the region highlighted by a red circle in the optical images shown in the left column of Fig. 3. Initially the analysis was performed under atmospheric conditions, where the headspace of the sample was saturated with air, then consecutive H_2_ and Ar gas flows were introduced in the cell. H_2_ gas at a flow rate of 250 sccm was introduced in the cell, while Ar gas flow rate was kept at 1000 sccm to make sure that any remaining H_2_ is removed from the cell. Both Ar and H_2_ gas streams were maintained in the cell for 2 hours. For the *in-situ* micro-Raman analysis, we used a laser spot of *ca*. 10 μm as shown in the supporting information (Fig. S3). The probed area of the sample on a 10x objective is shown with a red circle in the optical images (left panel). To better show the details of the probed region, a higher magnification optical image (100x) of the same position is also shown (middle panel). *In-situ* micro-Raman analysis under different controlled atmospheres are shown in the right panel. Micro-Raman analyses of the probed areas under ambient conditions show Raman shifts consistent with few layer thick deposits as discussed earlier.

Figure 3a shows the optical images and the corresponding *in-situ* micro-Raman analysis of MoSe_2_ deposits with sizes that are >10 μm. As shown in the optical image, since the size of the MoSe_2_ deposit is larger than the probed area. The basal plane of the deposit is probed under different atmospheres. Micro-Raman analysis of the >10 μm large sample reveals no shift in the position of the A_1g_ peak as the headspace of the cell is changed from air to H_2_, Ar and back to H_2_ respectively. Figure 3b shows the optical images and corresponding *in-situ* micro-Raman analysis of MoSe_2_ triangular deposits with sizes in the *ca*. 1–5 μm range. As shown in the optical images of the probed area of the film, multiple well-defined triangle-shaped deposits are present. AFM analysis shows that deposits are 1L-3L thick, *ca*. 1–3 nm (Fig. S4). Interesting, upon introducing H_2_ into the cell under mentioned conditions, a blue shift of *ca*. 1.8 cm^−1^ is observed. The observed shift is reversible. Passing Ar gas at 1000 sccm for 2 hours results in the same Raman shift as observed under ambient conditions, *ca*. 243.0 cm^−1^. The same blue shift of *ca*. 1.8 cm^−1^ appears upon introducing H_2_ at 250 sccm of flow (data is shown after 2 hours under the same conditions). Figure 3c shows the optical images and *in-situ* micro-Raman analysis of MoSe_2_ submicron triangular deposits. Raman spectroscopy response of this sample is consistent with few layer thick MoSe_2_ deposits. Micro-Raman analysis of the deposits under the ambient conditions show a Raman shift of *ca*. 242 cm^−1^. Here, also upon introducing H_2_, under mentioned conditions, a blue shift of ca. 1.3 cm^−1^ is observed. Raman spectroscopy analysis of the sample following exposure to Ar gas at 1000 sccm for 2 hours shows a Raman shift of 242 cm^−1^, which was observed under air. The *ca*. 1.3 cm^−1^ blue shift appears again upon passage of H_2_ over the sample at 250 sccm. The observed switchable Raman response points to the activation of a subset of MoSe_2_ deposits under H_2_ gas.

Similar blue shifts also appear when using flow rates as low as 100 sccm, however the waiting time required to observe a *ca*. 1.3 cm^−1^ blue shift in the position of the A_1g_ peak was *ca*. 4 hours (Fig. S5). The H_2_ induced changes in the position of the A_1g_ peak of the MoSe_2_ are also removed after exposure to atmospheric conditions for *ca*. 12 hours, as shown in Fig. S6. Lack of any blue shift in the presence of H_2_ in the deposit of the Fig. 3a can be attributed to the fact that no ultra-thin MoSe_2_ edge site is exposed to activate H_2_.

Our micro-Raman analysis provides *in-situ* spectroscopic evidence for hydrogenation of ultrathin MoSe_2_ deposits in the presence of H_2_ gas at room temperature. The observed spectroscopic response points to the presence of active sites for hydrogen chemisorption/release through the H_2_ (g) ⇋ H_ad_ + H_ad_ reaction, known as Tafel reaction. This step is particularly important in the catalytic pathways of hydrogen reactions. *In-situ* observation of hydrogen activation on dichalcogenide type materials, including MoSe_2_ are particularly interesting, since it provides insight into the nature of the active site for hydrogen reaction.

The analysis presented here clearly shows that hydrogenation of the MoSe_2_ is only present in few layer thick well defined triangular and rectangular deposits with exposed edge sites. The true nature and likely differences^18^ in activity of various active sites present on dichalcogenide type deposits is not addressed in this analysis, and future work using higher resolution and magnification in micro-Raman analysis may provide quantitative analysis of activity in different architectures toward hydrogen reactions.

## Conclusion

In summary we report on a simple and scalable vapor phase synthesis method for fabricating well defined MoSe_2_ deposits, where thickness, size and number density of the deposits can be controlled. Particularly, we show that well-defined 1–2 L MoSe_2_ deposits with submicron to few micron sizes can be grown on SiO_2_/Si substrates using this method. Our detailed characterization of the deposits using Raman spectroscopy and AFM analysis show and fully explains the interdependence of spectroscopic responses of CVD grown MoSe_2_ deposits with their thickness and size. Such array of MoSe_2_ deposits are then used for analyzing the reactivity of MoSe_2_ when exposed to H_2_ gas. We use Raman analysis of the A_1g_ peak of MoSe_2_ to probe reactivity of different architectures under reactive, H_2_ (g) and inert, Ar (g), conditions. Particularly, we provide *in-situ* spectroscopic evidence for hydrogen adsorption at select atomically thin MoSe_2_ deposits with exposed edge sites at room temperature. The observed spectroscopic response is not present when basal plane of large MoSe_2_ are probed. Our results provide insight into the activation and reaction mechanisms of hydrogen reactions on MoSe_2_ and other asymmetric dichalcogenides type materials.

## Methods

### Synthesis

MoSe_2_ (99.9% metals basis, Alfa Aesar) powder was used as received. Synthesis is carried out through vapor phase deposition. The synthesis environment is enclosed in a 1-inch diameter quartz tube, which was placed in a programmable tube furnace (Mini-Mite, Fisher Scientific). Copper pellets are placed upstream of the MoSe_2_ powder to remove any remaining oxygen coming from the Ar and H_2_ gas tanks. An alumina boat holds 0.10 g of MoSe_2_ powder and is placed closest to the thermocouple. The furnace was heated at a rate of 100 °C/h under a Ar-H_2_ mixture. Si/SiO_2_ (100) substrates were used for this synthesis. The environment was purged with 500 sccm Ar and 100 sccm H_2_ for one hour then kept under 500 sccm Ar for seven and half hours before deposition. The oven was then held at 850 °C for twenty minutes under the target flow rate of H_2_. Following deposition, the samples were cooled down to room temperature under Ar gas.

The Si/SiO_2_ substrates were cleaned by consecutive rinsing and sonication for 15 min with Milli-Q water, acetone and isopropanol respectively. A final rinsing with Milli-Q was performed prior to drying the wafers with compressed air.

### Optical images

Optical images of the deposits were obtained following synthesis using an Olympus light microscope and objectives. The optical images are used to assess the type and shape of the deposits made.

### Raman spectroscopy

Raman analysis was performed using a Horiba LabRAM HR Raman Spectrometer. A 532 nm excitation is used with a 600 mm^-1^ grading. Laser power was kept at *ca*. 3.1 mW for 532 nm to prevent burning. The setup provides a resolution of 0.1 cm^−1^.

### Atomic force microscopy

A Bruker MultiMode AFM instrument was used. A chromium cantilever was used to scan a A10 μm × 10 size area with a drive frequency of 246 kHz and a scan frequency of 0.3 kHz.

### *In-situ* analysis of hydrogen interactions with MoSe_2_ deposits

MoSe_2_ nanocrystals were placed in an air sealed reaction cell with a quartz window, which its headspace was saturated with mentioned gases using digital flow meters. The samples were first analyzed under air and then under different gas environments.

## Supplementary information

Supplementary information.
