# Supplementary material for: In-situ Raman analysis of hydrogenation in well-defined ultrathin molybdenum diselenide deposits synthesized through vapor phase deposition
Source: Sci Rep. 2020 Jun 23;10:10190. doi: 10.1038/s41598-020-67132-0 (PMC7311385; doi:10.1038/s41598-020-67132-0)
Supplement: Supplementary file 1 — Supplementary information. [file 41598_2020_67132_MOESM1_ESM.docx]

***In-situ* Raman analysis of hydrogenation in well-defined ultrathin molybdenum diselenide deposits synthesized through vapor phase deposition**

Peter Santiago^1^, Francisco Ramirez^1^, Hadi Tavassol*^2^

^1^Department of Physics and Astronomy, California State University, Long Beach

^2^Department of Chemistry and Biochemistry, California State University, Long Beach

*Correspondence to hadi.tavassol@csulb.edu

**Supporting information**

Schematic of the synthesis chamber:


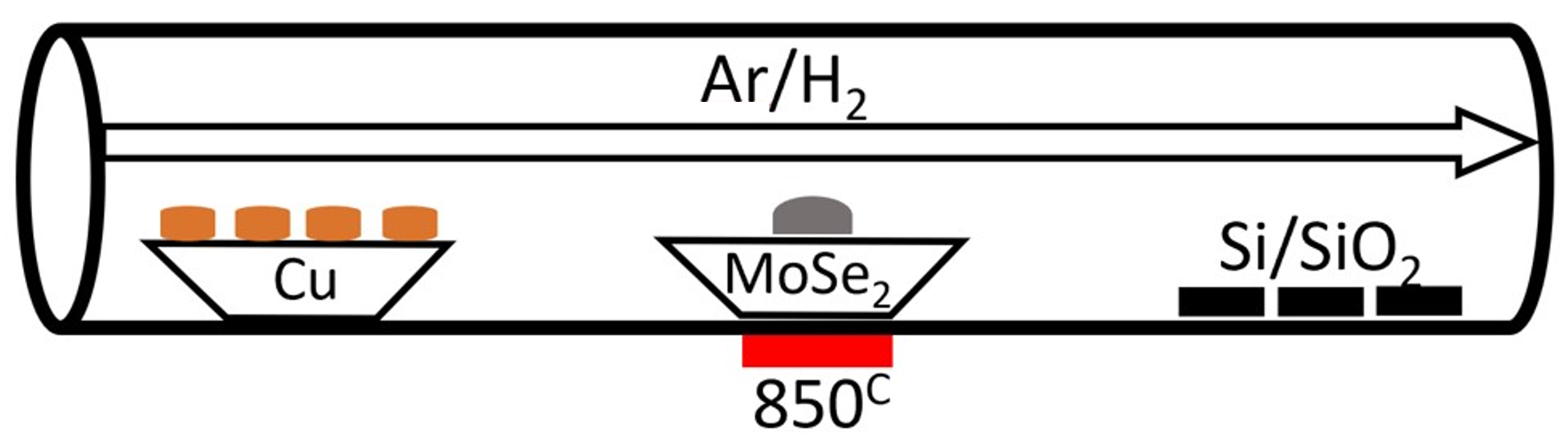


**Fig. S1** Schematic of the CVD set up with MoSe_2_ powder as the only precursor and heating. Table of applied gas flow rates and respective time frames at varying temperatures. 500 sccm Ar was critical in cleaning the synthesis environment prior to deposition temperature.

**Table S1.** Synthetic parameters for the vapor phase synthesis method described here.

| Temperature (°C) | Ar (sccm) | H_2_ (sccm) | Time (hours) |
| --- | --- | --- | --- |
| 30-100 | 500 | 100 | 1 |
| 100-850 | 500 | 0 | 7.5 |
| 850-850 | 0 | 20/75/100 | 10-20 mins |
| 850-30 | 500 | 0 | Natural Cool Down |


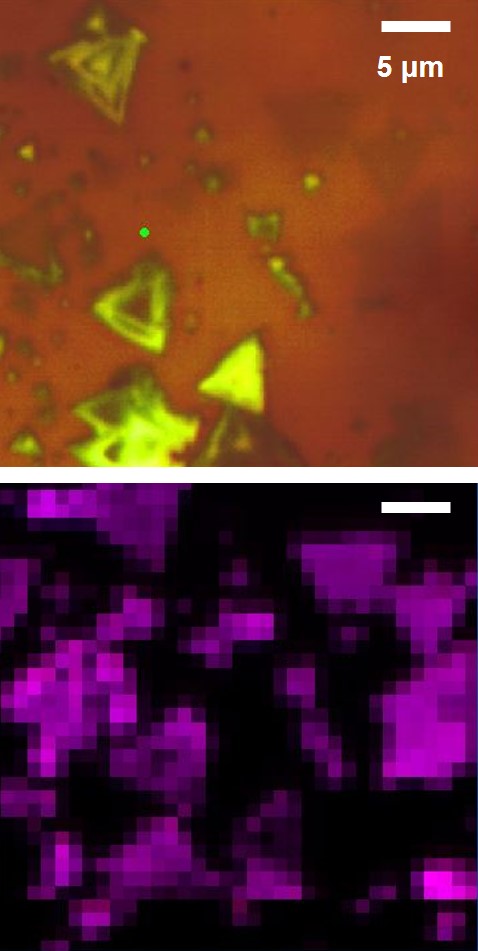


**Fig. S2** Optical image of MoSe_2_ triangles (top) and corresponding Raman mapping of the A_1g_ feature (bottom). The darker MoSe_2_ triangle (top) display brighter contrast in the Raman mapping (bottom) which displays thinner MoSe_2_ deposits. The brighter MoSe_2_ regions in the optical image (top) as the brighter regions have a darker contrast (bottom).


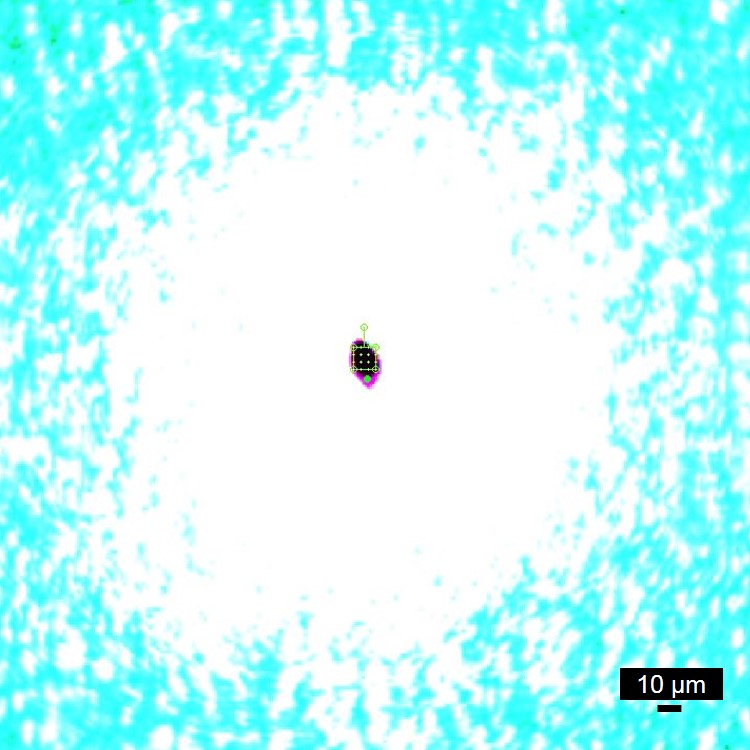


**Fig. S3** Optical image of the laser spot with a 10µm diameter used for the *in-situ* micro Raman analysis A 10µm x 10µm grid is placed in the middle of the laser spot (dark) to verify the size of the analyzed laser spot.


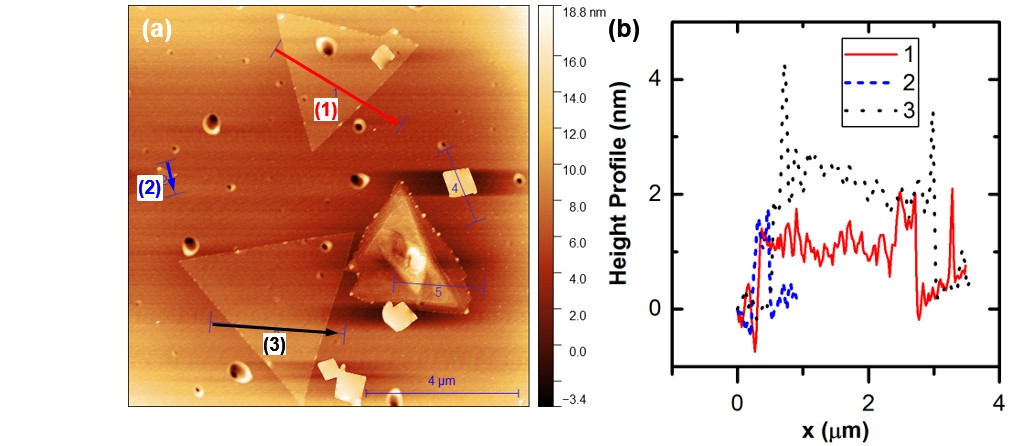


**Fig. S4** AFM analysis of the probed area from the figure 3 in the main text. Height profiles demonstrate mono to few layer MoSe_2_ deposits are grown via our synthesis method.

**
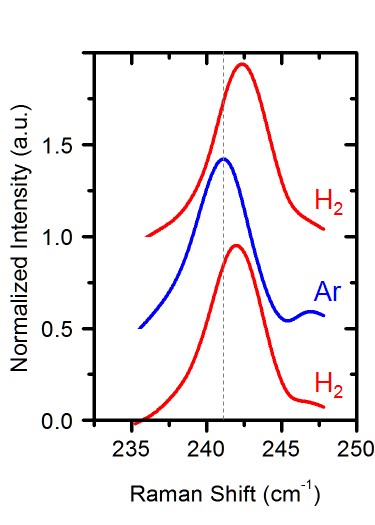
**

**Fig. S5** Raman shift changes in the A_1g_ region of MoSe_2_ deposits under 100 sccm of H_2_ for 4 hours (for both spectra labeled H_2_) and 500 sccm of Ar for 4 hours. A reversible behavior is observed after introducing H_2_ back into the cell following the Ar purging.

**
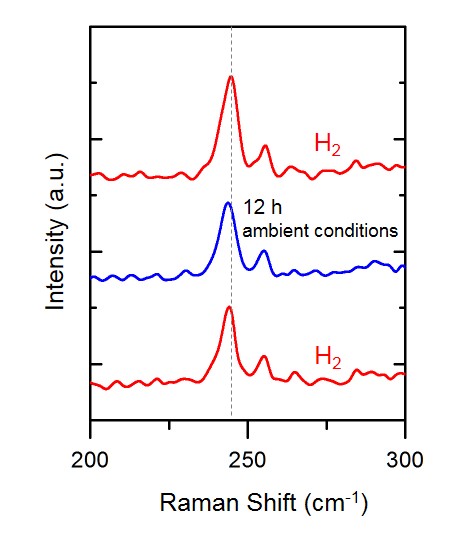
**

**Fig. 6S** Effect of overnight ambient conditions on H_2_ induced changes in the position of A_1g.­_


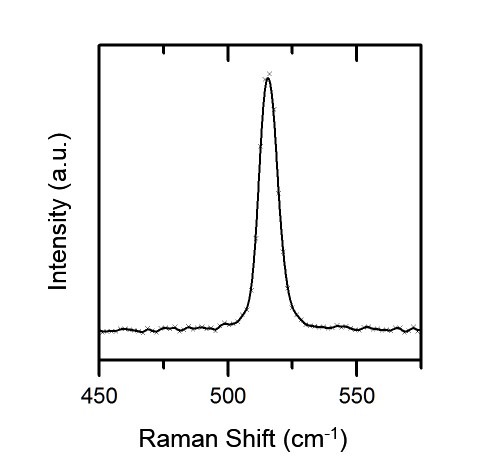


Fig. 7S Sample Raman spectrum of the SiO_2_/Si substrate used for the growth of the MoSe_2_ deposits.
